# Novel six-gene prognostic signature based on colon adenocarcinoma immune-related genes

**DOI:** 10.1186/s12859-022-04909-2

**Published:** 2022-10-11

**Authors:** Rui Zhou, Zhuowei Gao, Yongle Ju

**Affiliations:** 1 Surgical Department of Gastrointestinal Surgery, Shunde Hospital of Southern Medical University, No. 1 Jiazi Road, Shunde District, Foshan, 528399 Guangdong China; 2 Medical Department of Traditional Chinese Medicine, Shunde Hospital of Guangzhou University of Traditional Chinese Medicine, No. 12, Jinsha Avenue, Shunde District, Foshan, 510006 Guangdong China

**Keywords:** Colon adenocarcinoma, Immunotherapy, Cancer prognosis, Prognostic prediction model, Prognostic risk model, Immune-related genes

## Abstract

**Background:**

Colon adenocarcinoma (COAD) is one of the most common gastrointestinal tumors worldwide, and immunotherapy is one of the most promising treatments for it. Identifying immune genes involved in the development and maintenance of cancer is key to the use of tumor immunotherapy. This study aimed to determine the prognostic value of immune genes in patients with COAD and to establish an immune-related gene signature. Differentially expressed genes, immune-related genes (DEIGs), and transcription factors (DETFs) were screened using the following databases: Cistrome, The Cancer Genome Atlas (TCGA), the Immunology Database and Analysis Portal, and InnateDB. We constructed a network showing the regulation of DEIGs by DETFs. Using weighted gene co-expression network analysis, we prepared 5 co-expressed gene modules; 6 hub genes (*CD1A*, *CD1B*, *FGF9*, *GRP*, *SERPINE1*, and *F2RL2*) obtained using univariate and multivariate regression analysis were used to construct a risk model. Patients from TCGA database were divided into high- and low-risk groups based on whether their risk score was greater or less than the mean; the public dataset GSE40967, which contains gene expression profiles of 566 colon cancer patients, was used for validation.

**Results:**

Survival analysis, somatic gene mutations, and tumor-infiltrating immune cells differed significantly between the high- and low-risk groups.

**Conclusions:**

This immune-related gene signature could play an important role in guiding treatment, making prognoses, and potentially developing future clinical applications.

**Supplementary Information:**

The online version contains supplementary material available at 10.1186/s12859-022-04909-2.

## Background

Colorectal cancer (CRC) is one of the most common digestive system malignancies, accounting for approximately 1.2 million new cases and 600,000 deaths every year worldwide [1]. More than 2.2 million new CRC cases and 1.1 million deaths are projected in 2030 [2]. Colon adenocarcinoma (COAD) is the most common histopathological type of CRC. It often evolves into invasive cancer due to gene mutations and continuous accumulation of colonic adenomatous lesions [3]. Current treatments for patients with CRC include surgery, radiation, chemotherapy, targeted therapy, and immunotherapy. The 5 years survival rate exceeds 90% who undergo curative surgery for patients with localized tumor [4]. However, most patients are in the middle to late stages of the disease when diagnosed, in which case the 5 years survival rate decreases to approximately 10% [5]. The resistance of cancer cells to the immune response has been recognized as a new sign of cancer, and in recent years specific immune checkpoint therapeutic techniques have been extensively investigated [6]. Immune checkpoint inhibitors (ICIs), which manipulate the immune system to reactivate the antitumor immune response by blocking immune checkpoint proteins (PD-1 and CTLA-4) or their ligands (PD-L1), have been shown to have significant therapeutic effects in several cancers. However, immunotherapy does not achieve better efficacy in all CRC patients. Thus, it is equally important to understand the tumor immune microenvironment to explore tumor-associated immune signature biomarkers [7]. Moreover, characterizing immune function in different responding populations could help improve the efficacy of immunotherapy on CRC.

It has been reported that polygenic prediction models possess better predictive ability than single gene models for cancer prognosis. There have been a growing number of recent articles about immune-related genes predicting the risk of colon cancer. Ma et al. reported a prognostic model based on 13 immune-related genes [8], Wang et al. [9] also identified a novel prognostic signature of immune-related genes for CRC patients. A different prognostic model based on another 13 immune-related genes was recognized by Wang’s group [10]. However, the complexity and diversity of the data and the way in which this information can be used effectively is a formidable challenge. Methods for online public datasets analysis have not been standardized to date, so here we also provide our results for readers to compare. The combination with other similar predictive models will contribute to explain the predictive role of immune-related genes in COAD prognosis more comprehensively.

Accordingly, exploring the underlying connection between immune-related genes involved in the development and progress of COAD may conduce to build a prognostic prediction system for COAD. First, we used cancer patient information from the TCGA [11], ImmPORT, InnateDB, and Cistrome online databases to identify 6 immune-related genes that are associated with the prognosis of COAD patients. Next, we incorporated these six genes into the study accordingly to establish a risk model that predicts the survival prognosis of patients with COAD. Based on risk scores, we successfully divided COAD patients into low-risk and high-risk groups, which not only had different COAD prognosis, but also exhibited different gene expression profiles and different tumor-infiltrating immune cell characteristics. Targeting immune-related genes can deepen our understanding of the role that tumor immunity plays in COAD. This risk assessment model could provide more guiding significance for predicting the prognosis and even precision treatment of COAD patients.

## Results

### Data processing and identification of differentially expressed immune-related genes (DEIGs), genes (DEGs), and transcription factors (DETFs)

**Fig. 1:**
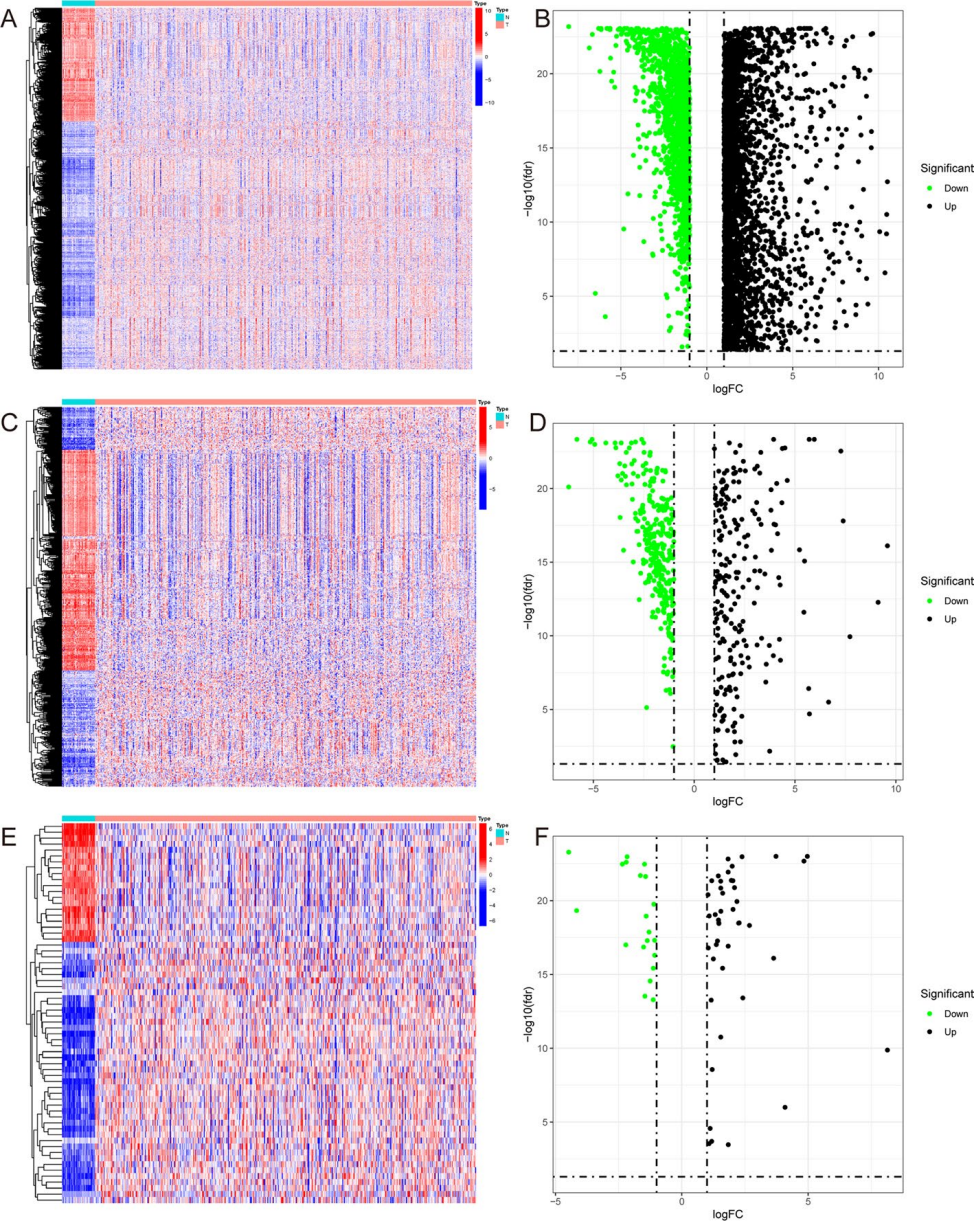
**A** Heatmap of 7782 differentially expressed genes (DEGs) in COAD tissues and normal tissues from the TCGA database. **B** Volcano plot of DEGs. **C** Heatmap of 649 differentially expressed immune-related genes (DEIGs) in COAD tissues and normal tissues from ImmPORT and InnateDB databases. **D** Volcano plot of DEIGs. **E** Heatmap of 67 differentially expressed transcription factors (DETFs) from Cistrome Project. **F** Volcano plot of DETFs

The transcriptome RNA sequencing data and clinical materials of 514 patients with COAD were obtained from TCGA. The screening criteria for DEGs between tumors and normal samples was set as a false discovery rate (FDR) < 0.05 and |log fold change (FC)|> 1, a total of 7782 differentially expressed genes were identified (Fig. 1A), B). A list of 2660 immune genes was obtained from the Immunome database, which were downloaded from InnateDB and ImmPORT databases; a total of 649 DEIGs were obtained from the screening (|log FC|> 1, FDR < 0.05) (Fig. 1C, D). We also obtained 318 transcription factors (TFs) from the Cistrome program and 67 DETFs from screening (Fig. 1E, F).

### Functional analysis of DEIGs

**Fig. 2:**
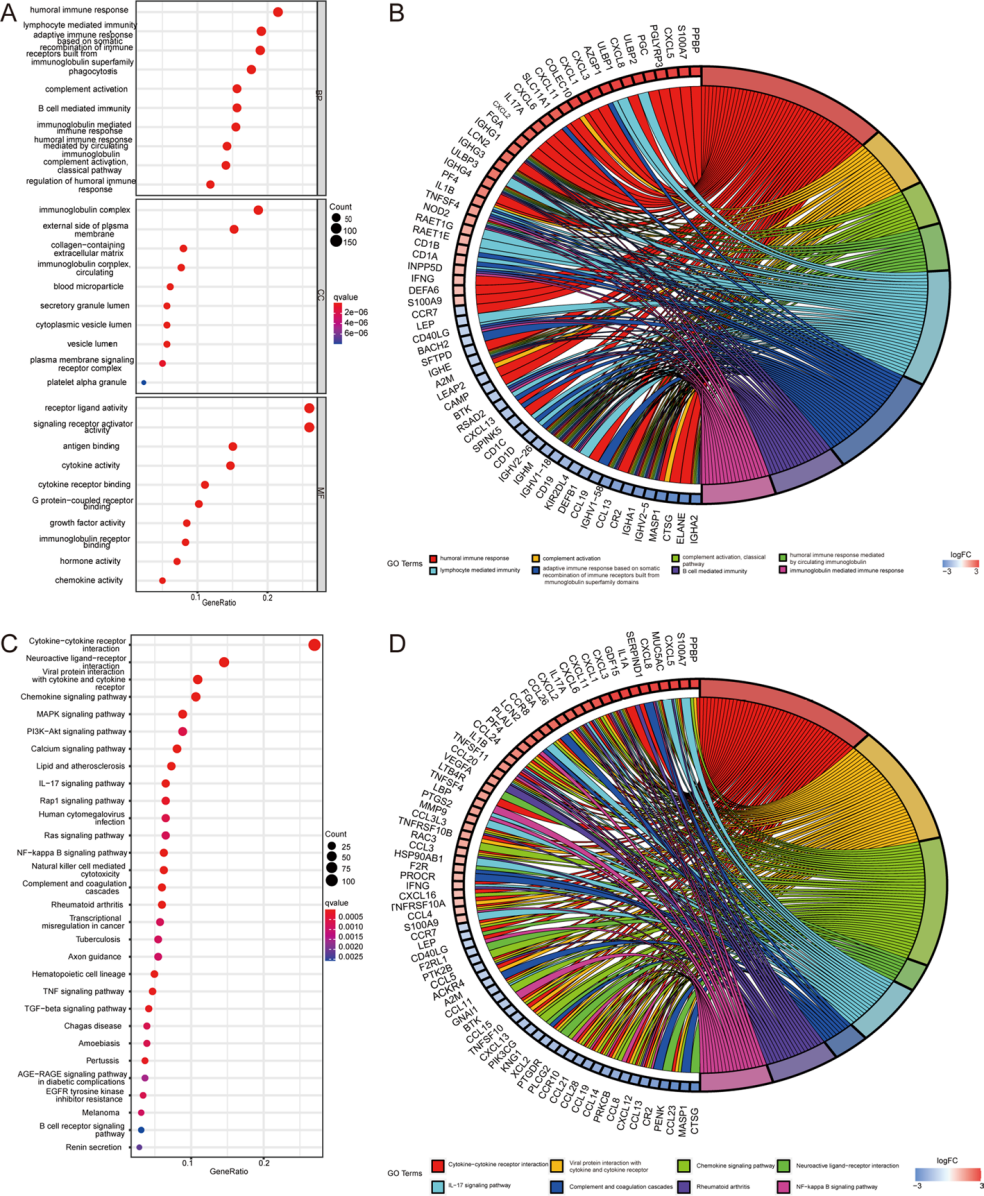
**A** Bubble diagram of the gene ontology (GO) enrichment analysis of DEIGs. **B** Circle diagram of the GO enrichment analysis of DEIGs. **C** Bubble diagram of the Kyoto Encyclopedia of Genes and Genomes (KEGG) enrichment analysis of DEIGs. **D** Circle diagram of the KEGG enrichment analysis of DEIGs

Gene ontology (GO) and Kyoto Encyclopedia of Genes and Genomes (KEGG) enrichment analyses of the DEIGs were conducted. The GO-biological process (BP) analysis indicated that the DEIGs were mainly involved in B cell immunoglobulin-mediated immune responses, complement activation, and regulation of immune system processes (Fig. 2A, B). The KEGG analysis indicated that the DEIGs were mainly involved in cytokine–cytokine receptor interaction, viral protein interaction with cytokines and cytokine receptors, chemokine signaling pathways, the IL-17 signaling pathway, neuroactive ligand–receptor interactions, and the nuclear factor (NF)-κ B signaling pathway (Fig. 2C, D).

### Building the DEIG and DETF interaction network

**Fig. 3:**
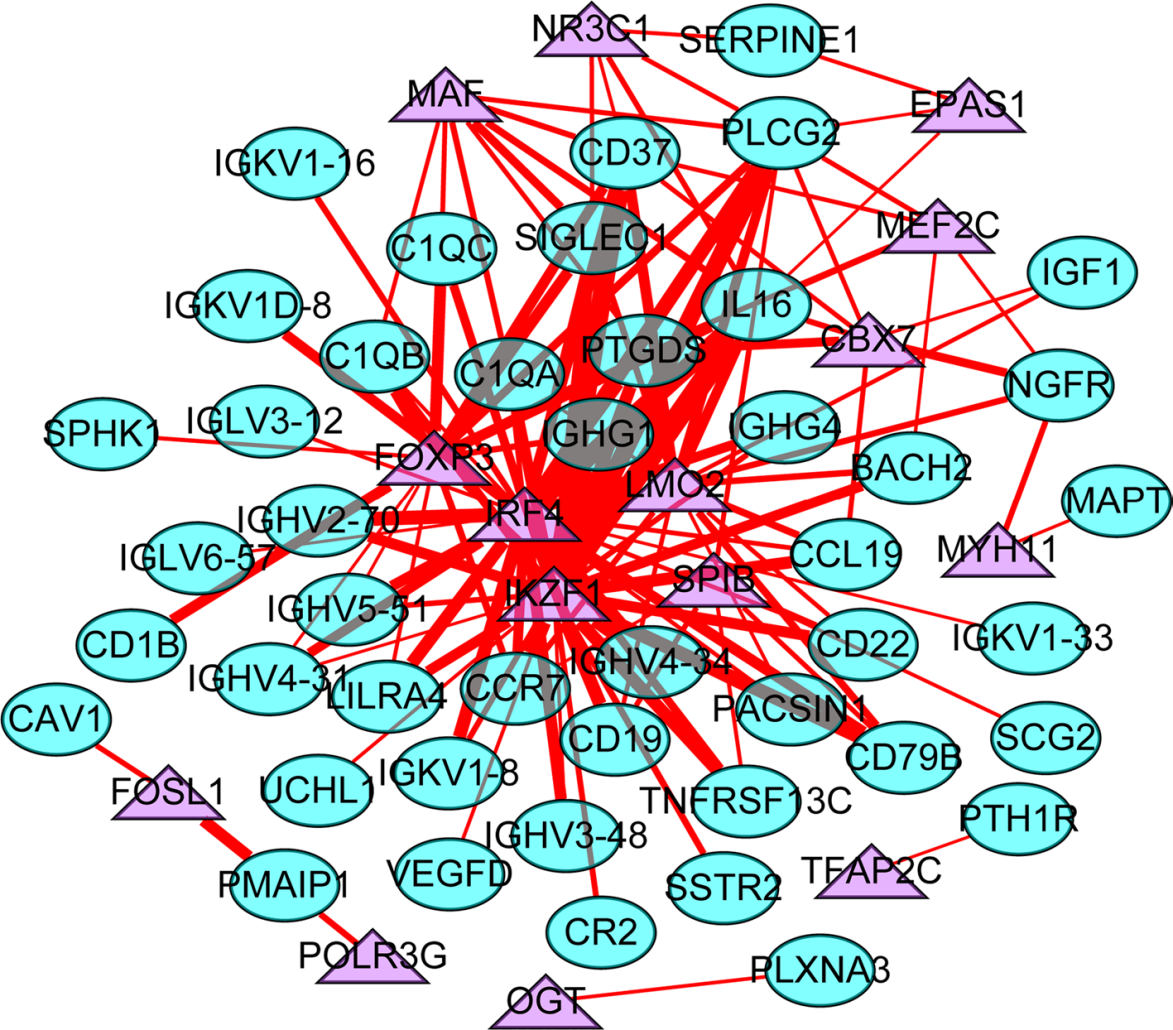
Regulatory networks between DEIGs and DETFs. Green circles represent DEIGs, purple triangles represent DETFs, and red lines represent positive regulation. The thicker edge represents the stronger the correlation between DEIGs and DETFs

The correlation between DEIGs and DETFs was obtained using these screening criteria: cor > 0.5 and p < 0.001. The correlation network diagram was drawn using Cytoscape (Fig. 3). The specific correlation results are shown in Additional file1: Table S1.

### Weighted gene co-expression network analysis (WGCNA) of DEIGs

**Fig. 4:**
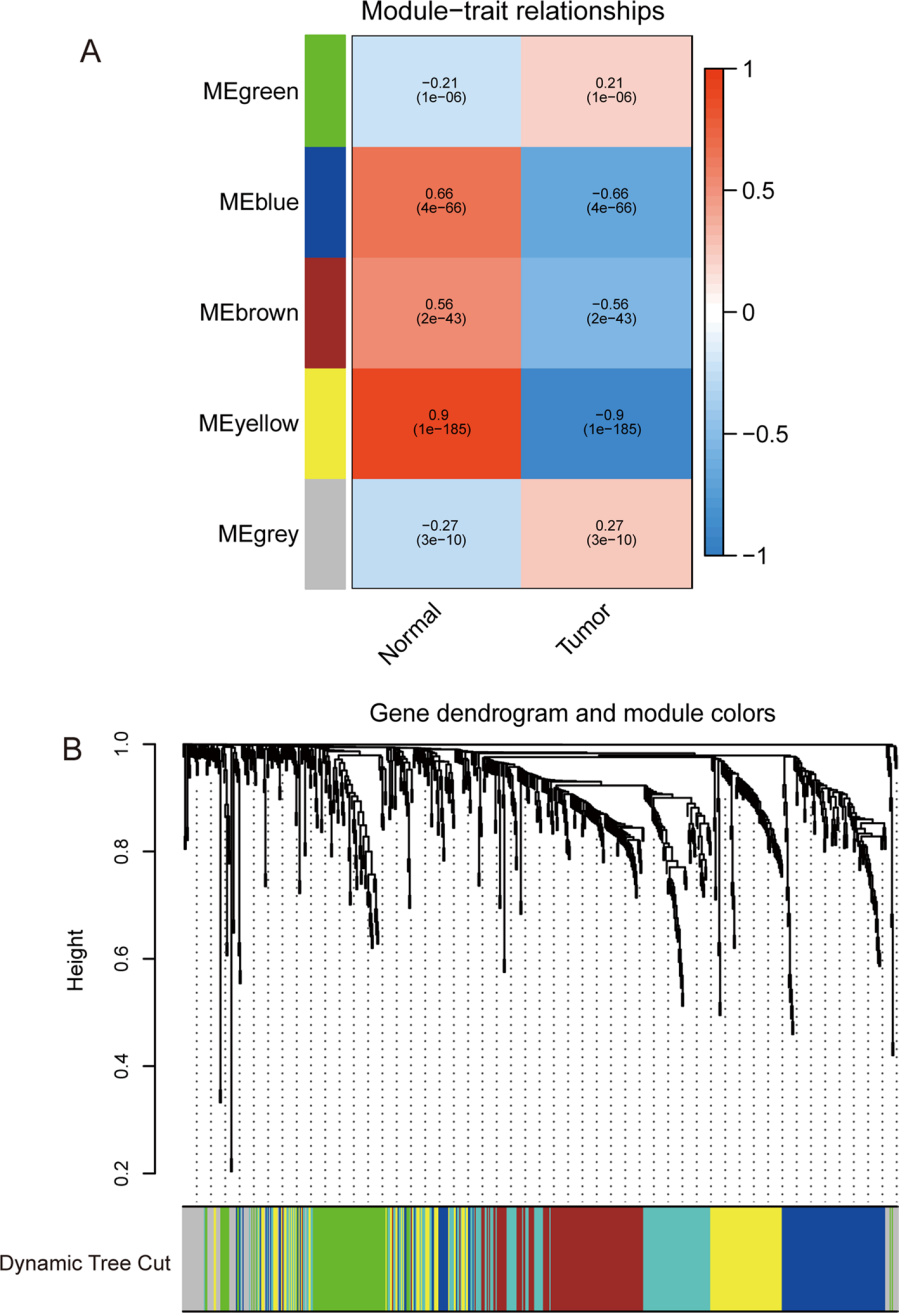
**A** Weighted gene correlation network analysis (WGCNA)-identified five gene co-expression modules (“MEgreen,” “MEblue,” “MEbrown,” “MEyellow,” and “MEgrey”). **B** Hierarchical clustering dendrogram of DEIGs. Different colors represent highly connected gene modules containing a cluster of functionally related genes

WGCNA [12, 13] identifies gene modules with similar expression patterns by calculating gene expression relationships, analyzing relationships between gene modules and phenotypes, and mapping the regulatory network between genes in the gene module and central genes (hubs). The WGCNA package in the R software was used to divide DEIGs into five modules (“MEgreen,” “MEblue,” “MEbrown,” “MEyellow,” and “MEgrey”) (Fig. 4). The optimal power value was 4. Prognostic models were built based on the minimal p-value (< 0.05). Genes in the green module were selected for subsequent analysis.

### Obtaining immune genes related to the prognosis of COAD patients

**Fig. 5:**
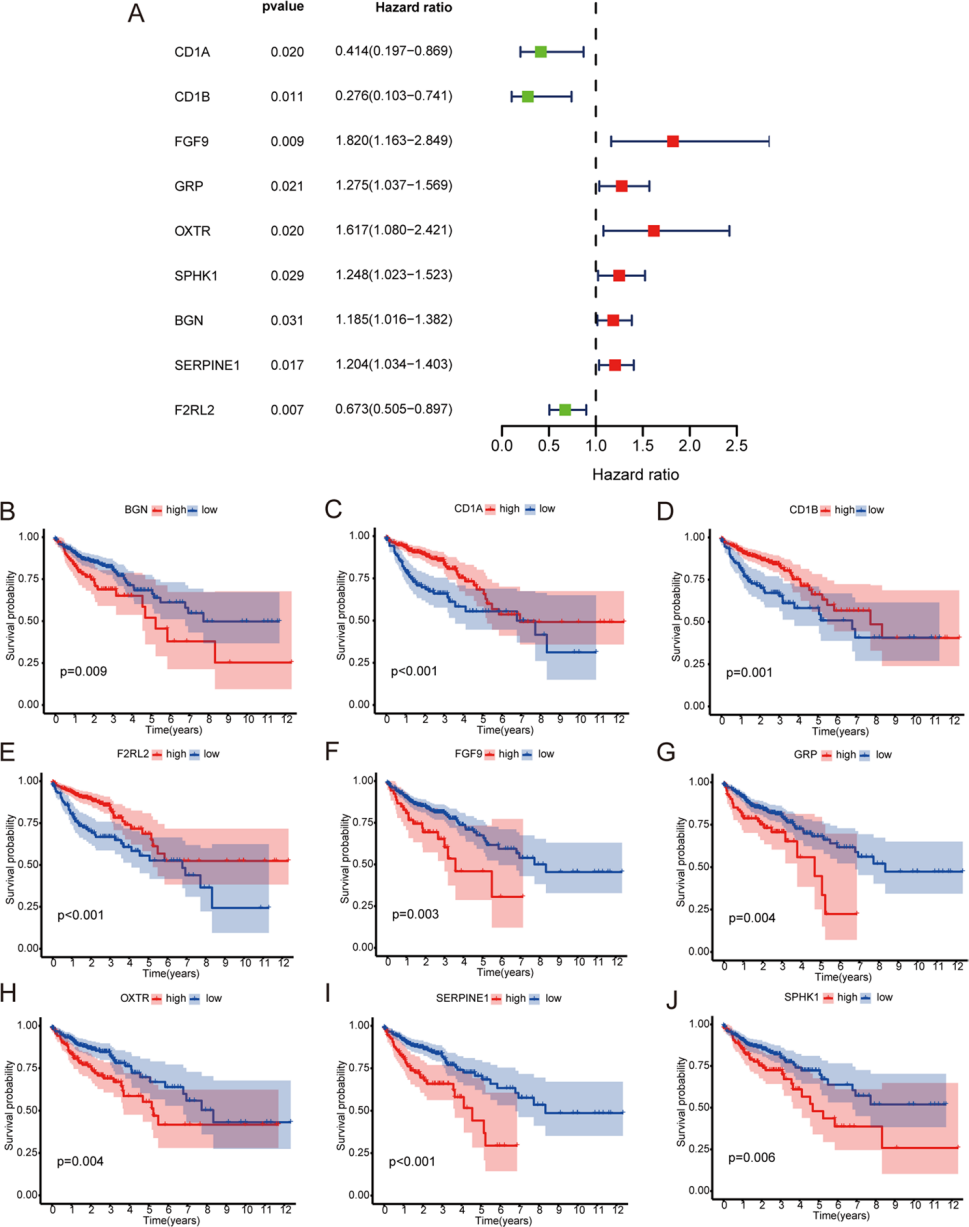
**A** Forest map of univariate regression analysis of 9 immune-related immune genes from MEgreen module in WGCNA analysis. **B**–**J** The K–M curve of 9 independent immune-related immune genes from MEgreen module in WGCNA analysis

After obtaining the “MEgreen” gene module, univariate regression analysis was performed on the clinical data of COAD patients in the TCGA and Gene Expression Omnibus (GEO) databases. Nine genes were screened as prognosis-related genes (*CD1A, CD1B, FGF9, GRP, OXTR, SPHK1, BGN, SERPINE1,* and *F2RL2*) using p < 0.05 as the selection criterion. The results of univariate analysis are shown in Fig. 5A. The Kaplan–Meier (K–M) curves of nine genes in COAD patients were plotted using the Survival package in R software (Fig. 5B–J).

### Building of a survival prognosis model and performing a survival analysis

**Fig. 6:**
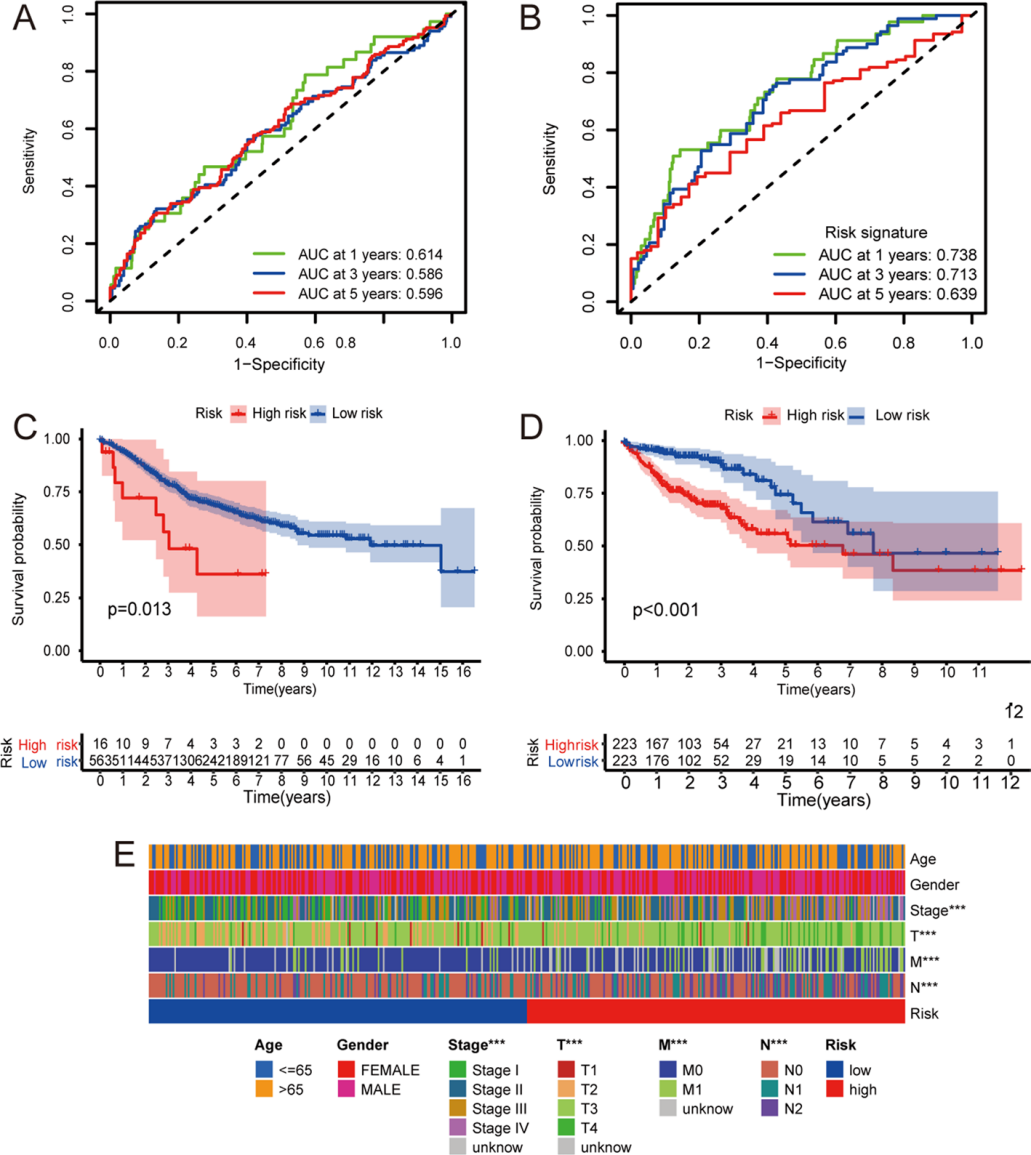
**A**, **C** Kaplan–Meier (K–M) analysis and ROC curves at 1, 3, 5 years of high- and low-risk groups in the GEO database. **B**, **D** K–M analysis and ROC curves at 1, 3, 5 years of high- and low-risk groups in TCGA database. **E** Clinicopathologic features including age, gender, stage, T, N, and M in high- and low-risk groups was shown in the heatmap

**Fig. 7:**
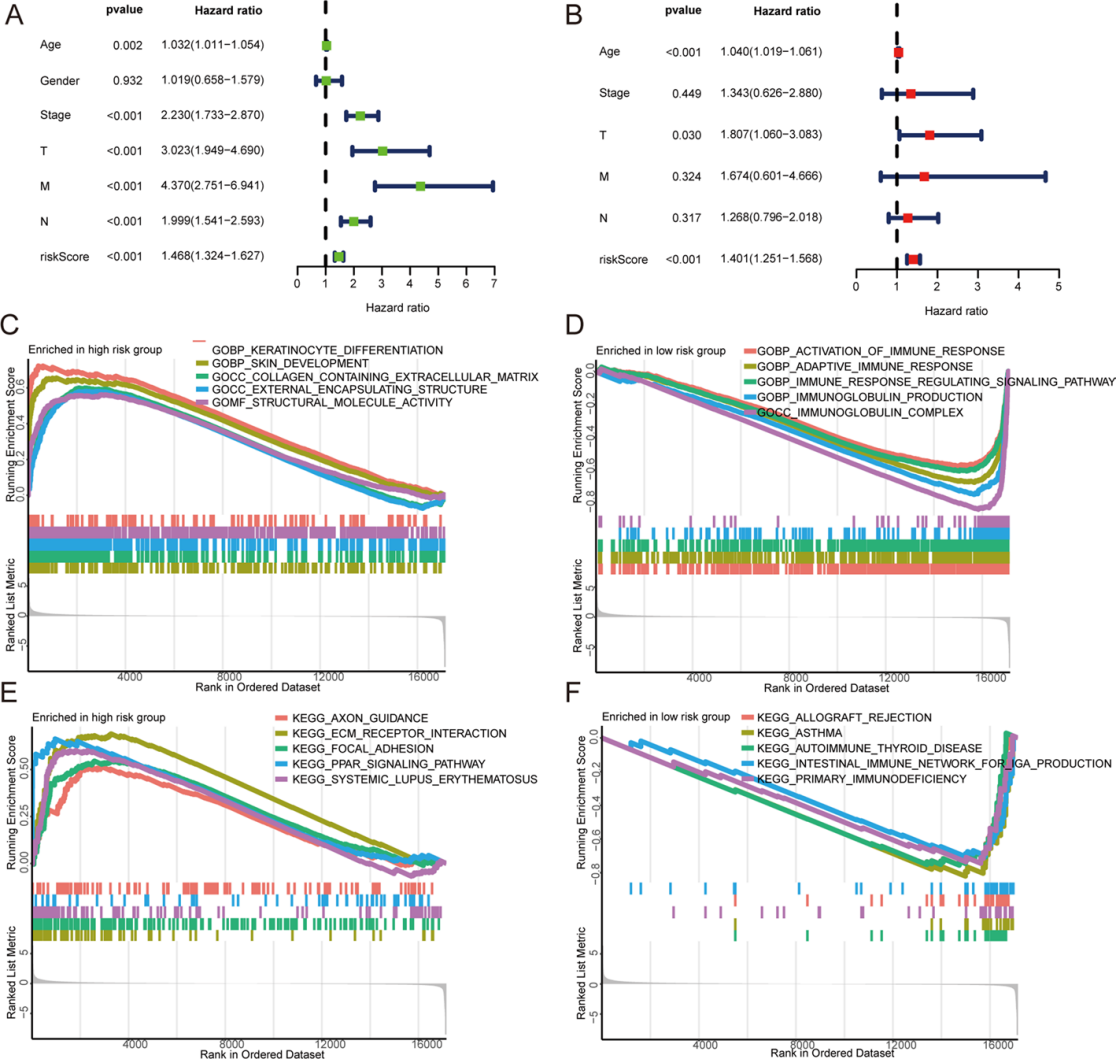
**A**, **B** Univariate and multivariate Cox regression analysis of high-risk and low-risk groups. **C**, **D** GSEA on GO enrichment analyses of high-risk and low-risk groups. **E**, **F** GSEA on KEGG enrichment analyses of high-risk and low-risk groups

The nine prognosis-related genes were included in a multivariate Cox regression analysis. The inclusion criteria were p < 0.05 and HR ≠ 1. Six genes (*CD1A*, *CD1B*, *FGF9*, *GRP*, *SERPINE1*, and *F2RL2*) were finally incorporated into the core model for risk calculation. The relationship between the genes and risk score is shown in Additional file2: Table S2. The composite risk scores of patients in TCGA datasets were calculated. The COAD patients were separated into low- and high-risk groups using the median risk score as the cutoff. The prognostic model was built using TCGA data and verified in the GEO datasets GSE40967. Further, we used the R software Survival and timeROC packages to draw two groups of K–M and receiver operating characteristic (ROC) curves, respectively (Fig. 6A–D), The R software ComplexHeatmap package was used to conduct a chi-square test between the demographics of the low- and high-risk groups and draw the clinical correlation heat map (p < 0.05, Fig. 6E). There were significant differences between the two groups in the tumor (T), node (N), metastasis (M) and stages. Univariate and multivariate Cox regression analyses revealed that age, T, stage, and risk score were independent prognostic factors for patients with COAD (Fig. 7A, B).

### Single-sample gene set enrichment analysis (ssGSEA) for low- and high-risk groups

The GO and KEGG enrichment analysis files (c5.go.v7.4.smbols and c2.cp.kegg.v7.4.symbols) were downloaded from the GSEA database (http://www.gsea-msigdb.org/gsea/index.jsp). GO and KEGG enrichment analyses of the high- and low-risk groups were performed using the clusterProfiler and the org.Hs.eg.db packages in R. GO enrichment analysis of the high-risk group indicated the main enrichment was in keratinocyte differentiation, skin development, collagen-containing extracellular matrix (ECM) external encapsulating structure development, and structural molecule activity. The most significantly enriched KEGG pathways were axon guidance, ECM receptor interaction, focal adhesion, the peroxisome proliferator-activated receptor (PPAR) signaling pathway, and systemic lupus erythematosus. GO enrichment analysis of the low-risk group indicated the main enrichment was in the activation of immune responses, adaptive immune responses, immune response regulating signaling pathways, immunoglobulin production, and immunoglobulin complexes. The KEGG pathway indicated enrichment in allograft rejection, asthma, autoimmune thyroid disease, the intestinal immune network for immunoglobulin A (IgA) production, and primary immunodeficiency (Fig. 7C–F).

### Comparison somatic mutation in high- and low-risk groups

**Fig. 8:**
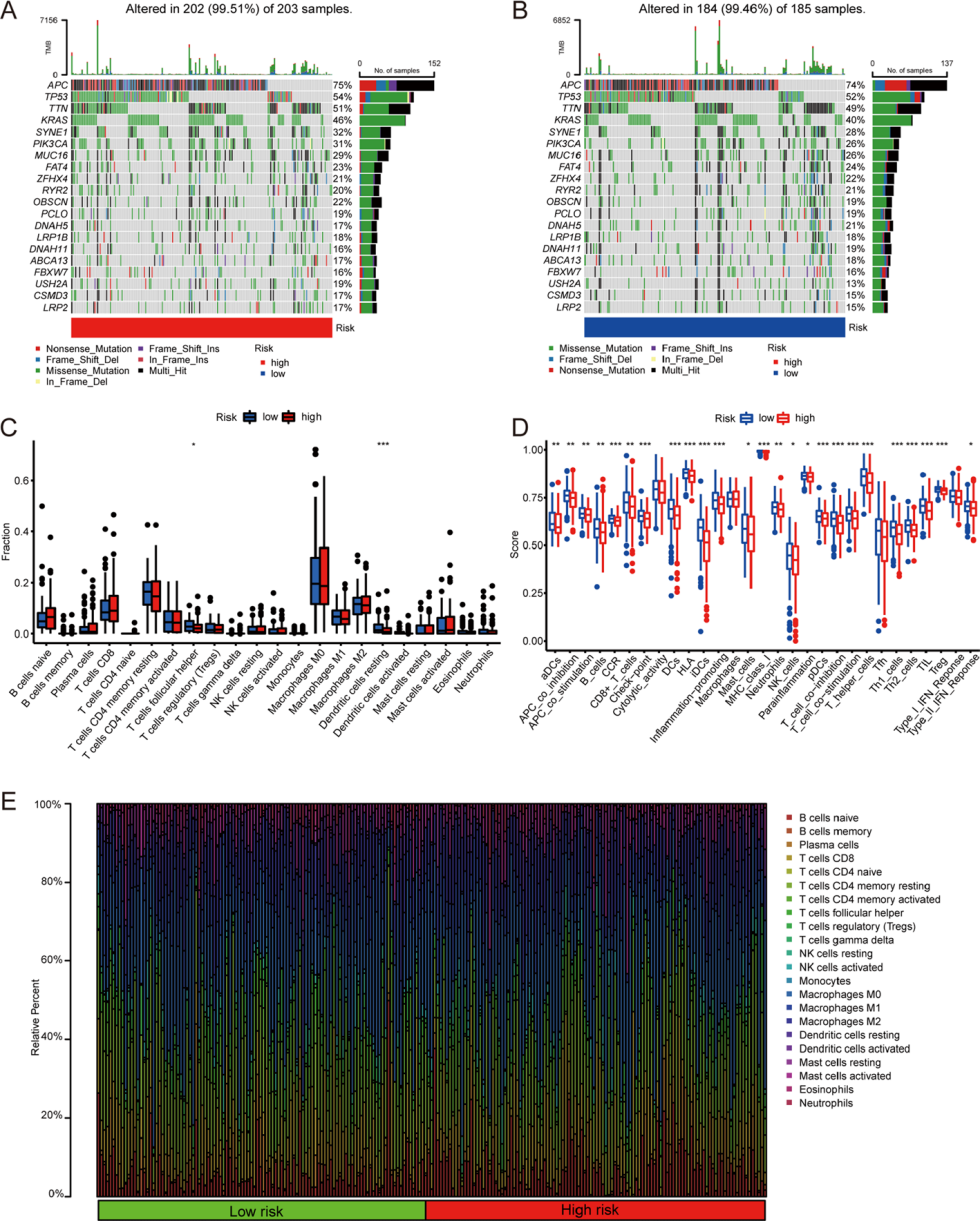
**A**, **B** Somatic mutation in high- and low-risk groups. **C** Differences in infiltrating immune cell distribution between high- and low-risk groups. **D** Differences in immune cell function between high- and low-risk groups. **E** Proportion of infiltrating immune cells in high- and low-risk groups

Somatic mutation profiles of patients with COAD downloaded from TCGA were analyzed and visualized using the R maftools package [14]. A total of 388 patients had mutations; after removing samples with no amino acid mutation, 202 were high-risk and 184 were low-risk. The five genes with the highest somatic mutation rate in the high-risk group were *APC*, *TP53*, *TTN*, *KRAS*, and *SYNE1*. Missense mutations were the most common category. The high‐risk group had a higher mutation frequency than the low‐risk group (Fig. 8A, B).

### Analyzing the tumor-infiltrating immune cells of high- and low-risk groups

Next, we used CIBERSORT to estimate the proportions of 22 distinct immune cell types (p < 0.05) (Fig. 8E), The Wilcoxon signed-rank test was used to determine the differences between tumor-infiltrating immune cells cells in the high-risk group. Resting dendritic cells and follicular helper T cells were present in significantly higher fractions in low-risk patients than in high-risk patients (p < 0.05; Fig. 8C). The immune cell function of the high-risk group was lower than that of the low-risk group with respect to adenomatous polyposis coli (APC) co-inhibition, APC co-expression, T-cell function, and macrophage function (Fig. 8D).

### Comparison with other models

**Fig. 9:**
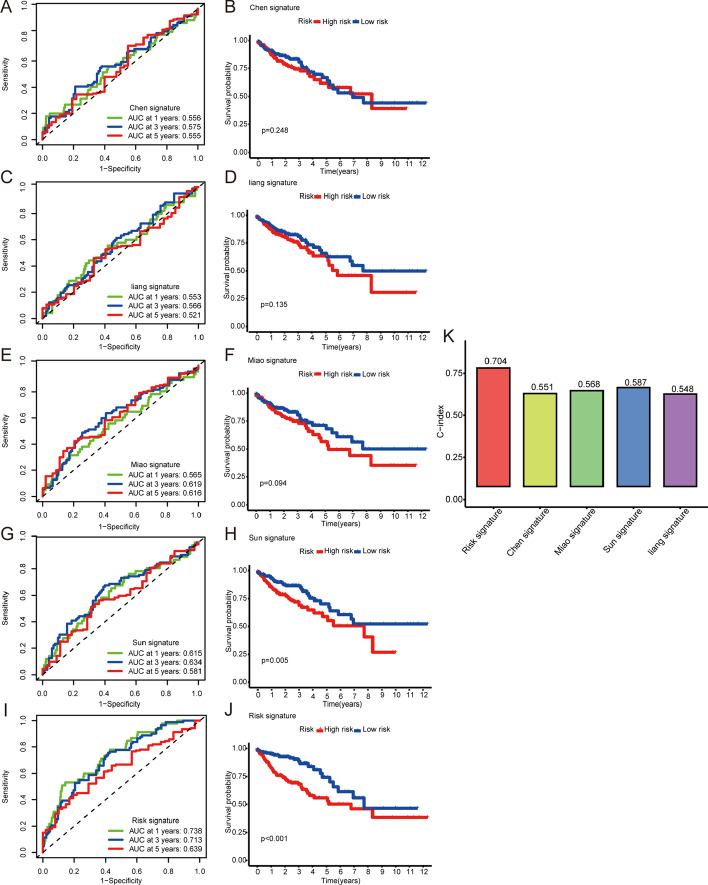
**A**, **B** 1, 3, and 5 years-time-dependent ROC and K–M curves of Chen’s signature. **C**, **D** 1, 3, and 5 years-time-dependent ROC and K–M curves of Liang’s signature. **E**, **F** 1, 3, and 5 years time-dependent ROC and K–M curves of Miao’s signature. **G**, **H** 1, 3, and 5 years time-dependent ROC and K–M curves of Sun’s signature. **K** Comparison of C-indexes of four published models with our risk model

We compared four corresponding prognostic models: a seven-gene signature (Sun), six-gene signature (Liang), twelve-gene signature (Mia), and seven-gene signature (Chen) [15–18]. We took the median risk value of all samples as the division standard, divided them into high- and low-risk groups, and used the four models to calculate the risk of the patients in TCGA. The ROC and K–M curves for the four models are shown in Fig. 9A–J. The values of area under the curve (AUC) for the four models at 5 years were 0.581, 0.521, 0.616, and 0.555, respectively, all significantly lower than our model (0.639). We calculated the concordance indexes (C-indexes), which were used to evaluate the prediction capability of the mixed-effect Cox model [20], of all models. The results showed that our model exhibited the highest C-index value (0.704; Fig. 9K).

### Verification of six immune-related genes in external databases

**Fig. 10:**
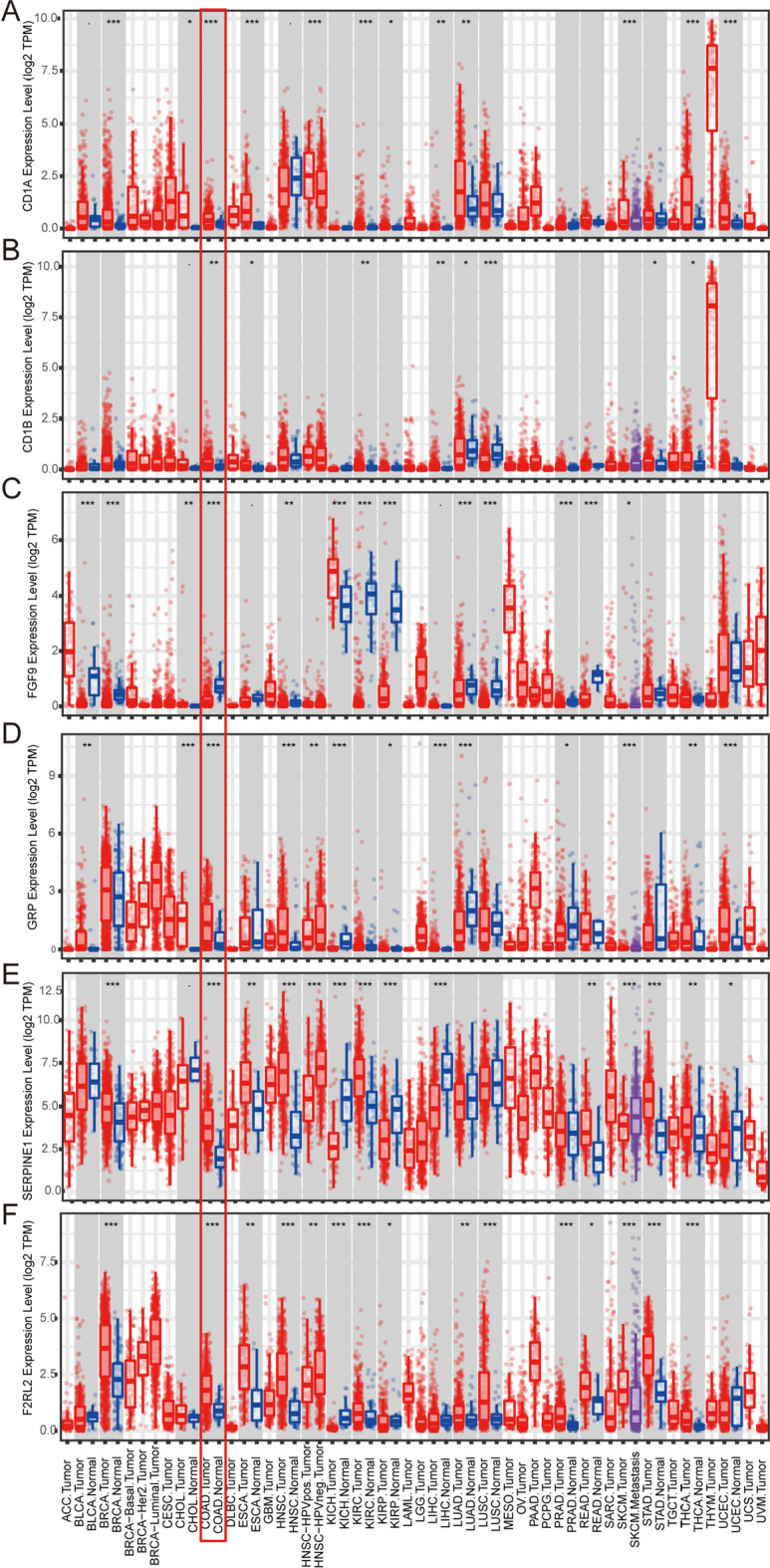
Gene expression profiles of 6 immune-related genes in this model in the Tumor Immune Estimation Resource (TIMER) database

**Fig. 11:**
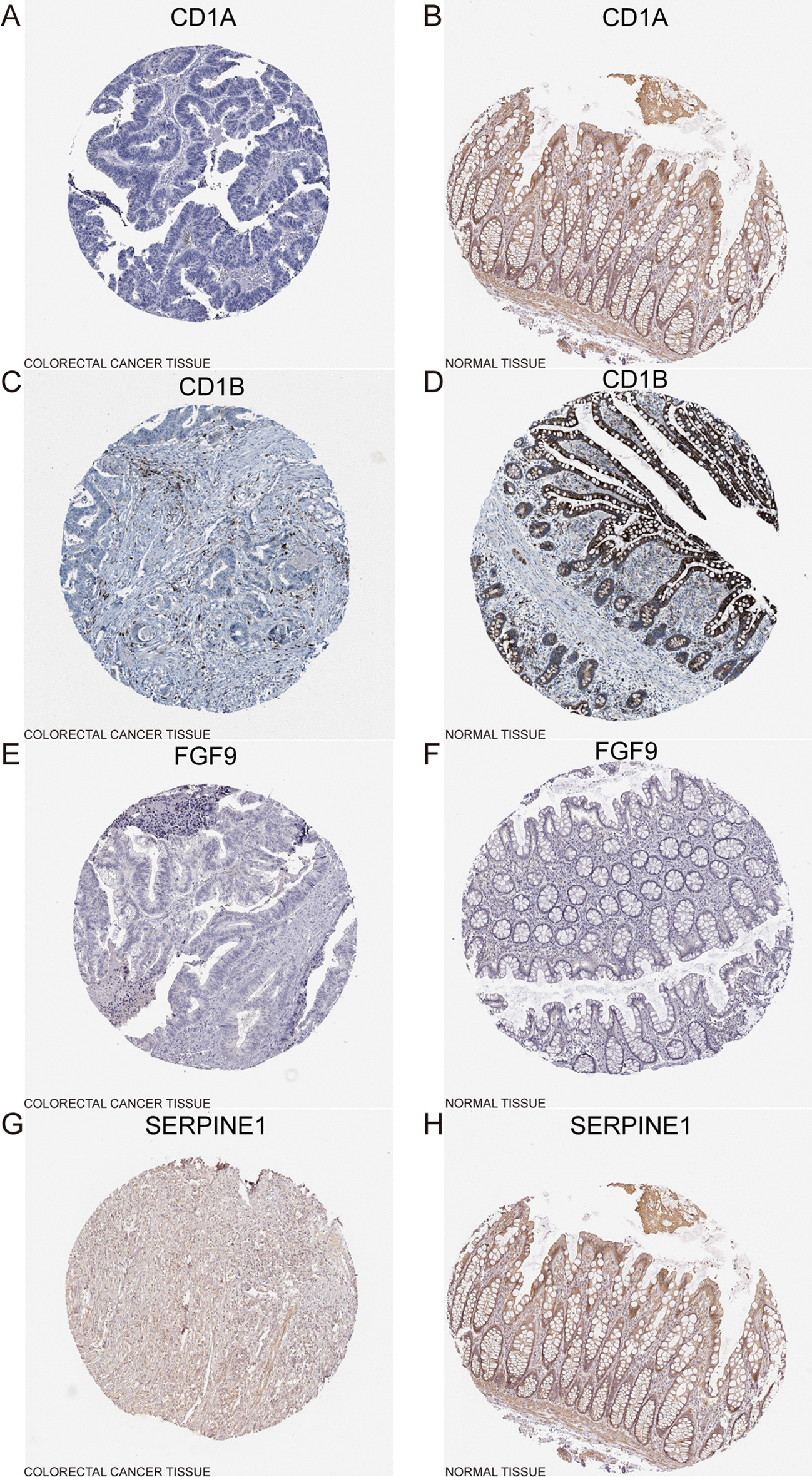
Differential protein expression of 6 prognostic genes in colon cancer and normal tissue using the HPA database (2F2RL2 were not included). **A**, **B** CD1A expression in colon cancer and normal tissue. **C**, **D** CD1B expression in colon cancer and normal tissue. **E**, **F** Fibroblast growth factor 9 (FGF9) expression in colon cancer and normal tissue. **G**, **H** SERPINE1 expression in colon cancer and normal tissue

The Tumor Immune Estimation Resource (TIMER) online database was used to analyze the differential expression of six genes in this model in 17 types of tumors and adjacent tissues. The *CD1A, CD1B, GRP, SERPINE1,* and *F2RL2* genes were highly expressed in tumor tissue. In contrast, *FGF9* was highly expressed in normal colon tissue (Fig. 10). A Human Protein Atlas (HPA) database search was performed to verify the protein expression levels of *CD1A, CD1B, SERPINE1,* and *FGF9* (Fig. 11).

## Discussion

Growing evidence suggests that the immune system plays a decisive role in the development and progression of CRC. FDA approval of anti-PD-1 monoclonal antibodies, as well as an anti-CTLA-4 monoclonal antibody, has improved the lives of some CRC patients, suggesting that immunotherapy had significant anticancer potential. However, immune checkpoint block could not achieve the desired response rate in all patients with CRC. This suggests that we still face many challenges in early diagnosis and treatment strategies for COAD, including a lack of awareness of high-risk patients, a lack of clinically applicable biomarkers, and the precise treatment for high-risk populations. Thus, we need to investigate the immune system intensively to understand how it interacts with cancer cells and describe the molecular characteristics of the COAD tumor immune microenvironment. More importantly, the research of predictive biomarkers may bring new expectations for the immunotherapy of CRC. Most studies focus only on a single molecule, but tumor development and metastasis are often the result of a synergy between multiple molecules. A single biomarker is difficult to achieve the specificity and sensitivity required in detecting cancer, current evidence suggests predictive models are more helpful than single biomarkers.

Therefore, in this current study, we developed a prognostic risk prediction model based on 6 immune-related genes. First, 7782 DEGs and 649 DEIGs were screened utilizing the TCGA database and InnateDB and ImmPORT databases. Next, WGCNA identified a set of DEIGs with similar expression patterns, and univariate and multivariate COX regression analyses confirmed that 6 of these DEIGs had independent prognostic value for patients with COAD. Then, these six hub genes (*CD1A*, *CD1B*, *FGF9*, *GRP*, *SERPINE1*, and *F2RL2*) were used to construct a risk score model to predict OS of COAD patients. Patients were divided into high- and low-risk groups, and the two groups exihibited completely different T, N, M, and stages. Moreover, GSEA analysis was used to derive differences in gene expression patterns between the high risk and low risk groups, meanwhile CIBERSORT assessment yielded different characteristics of tumor immune cell infiltration in the high- and low-risk groups. In the present risk prediction model, the higher the risk score, the worse the prognosis of the high-risk group and vice versa. The validation in the GEO database further confirmed the stability of this model. Thus, the successfully established immune-related gene model provides a new and effective method to predict the prognosis of COAD patients.

Currently, many studies have demonstrated that immune-related genes can coordinate and coordinate the onset and progression of cancer. CD1A is a lipid antigen-presenting molecule whose expression can be induced in monocytes and dendritic cells. CD1B is recognized as a marker by γδ T cells and plays an important role as an effector of tissue injury, infection, and cancer development [19]; it also regulates the differentiation and maturation of dendritic cells [20]. Fibroblast growth factor 9 (FGF9) plays a critical role in patients with colon cancer with resistance to epidermal growth factor receptor (EGFR)-targeted therapy, and combination therapy with anti-EGFR inhibitor may reverse drug resistance [21]. Gastrin-releasing peptide (GRP) may serve as an independent predictor of survival in patients with colon cancer [22]. SERPINE1 plays an essential role in remodeling the tumor microenvironment and the infiltration of immune cells [23]; some noncoding RNAs influence the epithelial–mesenchymal transition of colon cancer by regulating *SERPINE1* [24–26].

GO enrichment analysis of the high-risk group indicated the main enrichment was in keratinocyte differentiation, skin development, collagen-containing extracellular matrix (ECM) external encapsulating structure development, and structural molecule activity. Pradella et al. [27] revealed that Unc-5 netrin receptor B (UNC5B) is an axon guidance regulator whose expression is associated with tumor angiogenesis and poor prognosis. Mitra et al. demonstrated that cell migration involves integrating ECM with the actin cytoskeleton through transmembrane receptors [28]. Focal adhesion kinase (FAK) is activated by the ECM, promoting the formation of focal adhesion complexes and focal adhesion maturation [28–30]. Thus, the FAK signaling pathway may be activated in the high-risk group, leading to colorectal cancer invasion in vitro and metastasis in vivo. The most significantly enriched KEGG pathways were axon guidance, ECM receptor interaction, focal adhesion, the peroxisome proliferator-activated receptor (PPAR) signaling pathway, and systemic lupus erythematosus. In colon cancer, the absence of PPARα and PPARβ/δ expression promotes cancer growth, and PPARγ suppresses tumorigenesis through the regulation of and interaction with β-catenin [31–33]. Systemic lupus erythematosus is associated with abnormal autoimmune reactions; however, the mechanisms have not been identified. Colon cancer is one of the most common complications in gastrointestinal diseases [34]. Down-regulated molecular pathways in the low-risk group included allograft rejection, asthma, autoimmune thyroid disease, intestinal immune network for IgA production, and primary immunodeficiency. Asthma symptoms are alleviated by reducing eosinophil production; previous studies have proposed that the depletion of eosinophils due to asthma severely compromises antitumor immunity in syngeneic and genetic models of colorectal cancer. This association is possibly due to defective Th1 and CD8^+^ T-cell responses [35]. Bacteria are linked to cancer [36], and intestinal bacteria may make the tumor microenvironment more favorable for IgA production. Consequently, IgAs are widely used as biomarkers for early cancer screening [37–39]. Recent studies have indicated that patients with primary immunodeficiency tend to have a higher cancer incidence because of genomic instability due to defective DNA repair mechanisms [40, 41]. However, the association between autoimmune thyroid disease, allograft rejection, and colorectal cancer has not been found.

Overall, the current study introduces an immune-related gene module as a novel prognostic tool for COAD patients. The present study features a model based on a comprehensive population database and high-throughput sequencing data, which was successfully validated by subsequent testing in an external clinical cohort. Ultimately, identifying the immune function profile of high-risk populations can help improve the efficacy of immunotherapy for the precise treatment of CRC. Nevertheless, there are still some limitations that need to be mentioned. First, as different bioinformatics algorithms may lead to different results, the combination with other similar predictive models will contribute to explain the predictive role of immune-related genes in COAD prognosis more comprehensively. Second, transcriptome analysis does not reflect the molecular mechanisms of COAD immunobiology, which may be better elucidated by proteomics and/or metabolomics. Third, since this study is a retrospective study with statistics from online databases, the validation of predictive effectiveness of the model in clinical practice was required. Fourth, our predictive model can add predictive value to existing patient risk groupings. Combining risk scores, TNM systems and age synergistically or complementarily is essential for clinical work. In conclusion, although we initially explored the expression characteristics and immune associations of immune-related genes, these genes have not been fully elucidated and deserve further in-depth study. In the next work, we will continue to validate the prognostic accuracy of this model on a large scale with more samples and more external experiments.

## Conclusions

In summary, we created a six-gene prognostic model with good predictive capability in both the training and validation sets. This model could help clinicians predict individual risks of patients with COAD in the development of personalized COAD treatment.

## Methods

### Obtaining relevant data from network database

The gene expression and somatic mutation data of patients with COAD were obtained through the TCGA data portal (https://portal.gdc.cancer.gov/) (Additional files 3 and 4). Gene expression data and clinical information were downloaded from GEO databases (https://www.ncbi.nlm.nih.gov/geo/). The immune signatures were obtained from the ImmPORT (https://immport.niaid.nih.gov) and InnateDB databases (https://www.innatedb.ca/). The list of TFs was obtained from the Cistrome Project (http://www.cistrome.org/). Furthermore, the TIMER web tool (http://timer.cistrome.org/) was used to obtain the genes in the tumor microenvironment. The c5.go.v7.4.symbols and c2.cp.kegg.v7.4.symbols datasets were downloaded from MsigDB (http://www.gsea-msigdb.org/gsea/index.jsp) on the GSEA website.

### Identifying differentially expressed genes

Data extraction and integration were conducted using Perl (v5.32.1). DEIGs, DEGs, and DETFs were analyzed using R version 4.1.1 and the relevant Bioconductor packages (e.g., limma v3.48.3 and edgeR v3.34.1) [42] according to the screening criteria of |log2FC|> 1 and FDR < 0.05. Plots were generated using the R package ggplot2 (v3.3.5), and heatmaps were drawn using the R package pheatmap (v1.0.12).

### DEIGs enriched and analyzed using GO and KEGG

GO and KEGG enrichment analyses of screened genes were performed using the R package clusterProfiler. An FDR < 0.05 was set as the cutoff criterion to identify the outstanding GO terms and KEGG pathways visualized using bubble and circle diagrams.

### Correlation analysis between DEIGs and DETFs

Correlation analysis between DEIGs and DETFs was performed using the cor.test in R software (cor > 0.5, p < 0.001). Protein–protein interaction networks were generated using Cytoscape (version 3.8.2) [43].

### WGCNA of DEIGs

WGCNA was performed by applying the R package WGCNA to DEIGs to obtain a different module. It was used to generate the module network plots using the R igraph package. Subsequently, we determined the intersection of the module genes obtained from the GEO and TCGA databases.

### Immune-related immune genes obtained from intersection genes

Clinical information and gene expression for the univariate analysis were analyzed using Cox regression, and the corresponding DEGs were screened out as prognostic immune‐related genes for further study at p < 0.05 and |hazard ratio (HR)|≠ 1.

### Prognostic model construction

Based on the expression of screened genes from the previous step, a risk model was built using multivariate Cox regression model analysis, calculated as follows: Risk score = Expgene1 × coefgene1 + Expgene2 × coefgene2 + … + Expgenen × coefgenen. Exp represents the expression level of the gene and coef is the estimated regression coefficient of the gene derived from the multivariate Cox analysis.

### Evaluation and analysis of risk model

The RNA sequencing data of patients with COAD were obtained from TCGA database and set as the training set, whereas the external validation cohorts were obtained from the GEO dataset (GSE40967). The patients were then separated into high- and low-risk groups using the mean risk score in the training set as the cutoff value. Using the files downloaded from the MsigDB database, GO and KEGG enrichment analyses were performed for high-risk and low-risk groups using R software. K–M analysis and univariate and multivariate independent prognosis analyses were performed for the two groups using the survminer and survival R packages and the forest plot was drawn. The ROC curve and time-dependent ROC-based AUC were plotted using the R package timeROC. We found a similar prediction model in the literature and compared the predictive accuracy of the prognostic models using a C-index and plotting ROC curves to compare the AUC values.

### Analyzing somatic mutation in high- and low-risk groups

The tumor mutation burden (TMB) was calculated as mutations per megabase (mut/Mb). The tumor mutation of patients in the high and low-risk groups was analyzed using TMB data. The R package maftools was used to analyze and visualize the somatic mutation data.

### Correlation analysis of tumor-infiltrating immune cells

The proportion of infiltrating immune cells was calculated using the CIBERSORT algorithm [44] and the results were considered significant at p < 0.05. The Wilcoxon signed-rank test was used to analyze the differential abundance of infiltrating immune cells and immune cell function between the low- and high-risk groups, and box plots were created using the ggpubr package in R.

### Validation of reliability of risk models in other databases

The TIMER (https://cistrome.shinyapps.io/timer/) [45] database was used to verify the difference in gene expression between tumor and normal samples. Immunohistochemistry and the HPA (https://www.proteinatlas.org/) [46] database were used to compare protein expression between tumor and normal tissues.

## Supplementary Information

**Additional file1**. **Table S1**: Regulatory relationship between DEIGs and DETFs.

**Additional file2**. **Table S2**: DEIGs (multivariate Cox regression analysis) and regression coefficient.

**Additional file3**. TCGA clinical data.

**Additional file4**. TCGA data.

## Data Availability

The entire sequencing profile data and the clinical data of the patients with COAD in this study come from the TCGA (https://cancergenome.nih.gov/) and GEO databases (https://www.ncbi.nlm.nih.gov/geo/query/acc.cgi?acc=GSE40967). The TFs were obtained from the Cistrome Project (http://cistrome.org/CistromeCancer/CancerTarget/). The genes in the tumor microenvironment were obtained from the TIMER web tool (http://timer.cistrome.org/), and the immune-related genes were obtained from ImmPORT (https://www.immport.org/shared/genelists) and InnateDB databases (https://www.innatedb.ca/annotatedGenes.do?type=innatedb).

## Abbreviations

APC: Adenomatous polyposis coli
AUC: Area under the curve
BP: Biological process
COAD: Colon adenocarcinoma
CRC: Colorectal cancer
DEGs: Differentially expressed genes
DEIGs: Differentially expressed immune-related genes
DETFs: Differentially expressed transcription factors
ECM: Extracellular matrix
FAK: Focal adhesion kinase
GEO: Gene Expression Omnibus
GO: Gene ontology
GRP: Gastrin-releasing peptide
GSEA: Gene set enrichment analysis
HPA: Human Protein Atlas
KEGG: Kyoto Encyclopedia of Genes and Genomes
PPAR: Peroxisome proliferator-activated receptor
ROC: Receiver operating characteristic
TCGA: The Cancer Genome Atlas
TF: Transcription factor
TIMER: Tumor Immune Estimation Resource
TMB: Tumor mutation burden
WGCNA: Weighted gene co-expression network analysis
